# Gelam Honey Has a Protective Effect against Lipopolysaccharide (LPS)-Induced Organ Failure

**DOI:** 10.3390/ijms13056370

**Published:** 2012-05-23

**Authors:** Mustafa Kassim, Marzida Mansor, Nazeh Al-Abd, Kamaruddin Mohd Yusoff

**Affiliations:** 1 Department of Anesthesiology, Faculty of Medicine, University of Malaya, 50603 Kuala Lumpur, Malaysia; E-Mail: marzida@gmail.com; 2 Department of Biotechnology, Faculty of Science, University of Malaya, 50603 Kuala Lumpur, Malaysia; E-Mail: nazehali78@yahoo.com; 3 Department of Molecular Biology and Genetics, Faculty of Arts and Science, Canik Basari University, Samsun, Turkey; E-Mail: mykamar77@gmail.com

**Keywords:** honey, inflammation, lipopolysaccharide, rabbits, biochemical tests, myeloperoxidase

## Abstract

Gelam honey exerts anti-inflammatory and antioxidant activities and is thought to have potent effects in reducing infections and healing wounds. The aim of this study was to investigate the effects of intravenously-injected Gelam honey in protecting organs from lethal doses of lipopolysaccharide (LPS). Six groups of rabbits (*N* = 6) were used in this study. Two groups acted as controls and received only saline and no LPS injections. For the test groups, 1 mL honey (500 mg/kg in saline) was intravenously injected into two groups (treated), while saline (1 mL) was injected into the other two groups (untreated); after 1 h, all four test groups were intravenously-injected with LPS (0.5 mg/kg). Eight hours after the LPS injection, blood and organs were collected from three groups (one from each treatment stream) and blood parameters were measured and biochemical tests, histopathology, and myeloperoxidase assessment were performed. For survival rate tests, rabbits from the remaining three groups were monitored over a 2-week period. Treatment with honey showed protective effects on organs through the improvement of organ blood parameters, reduced infiltration of neutrophils, and decreased myeloperoxidase activity. Honey-treated rabbits also showed reduced mortality after LPS injection compared with untreated rabbits. Honey may have a therapeutic effect in protecting organs during inflammatory diseases.

## 1. Introduction

Honey is a natural, sweet and viscous fluid produced by bees from floral nectar, which comprises more than 400 different chemical compounds [1], including proteins, enzymes, organic acids, mineral salts, vitamins, phenolic acids, flavonoids, free amino acids, and small quantities of volatile compounds [2]. Historically, honey has been used as a treatment for a broad spectrum of injuries, including wounds, burns and ulcers [3,4]. Honey has also been reported to stimulate the immune system (monocytes, neutrophils) [5–7]. It also clears infection by boosting the immune system, exerting anti-inflammatory and antioxidant activities, and stimulating cell growth [8]. Gelam honey inhibits nitric oxide (NO) and cytokine release both *in vitro* and *in vivo* [9,10].

Lipopolysaccharide (LPS) stimulates innate immune responses that mediate the cellular release of NO and various proinflammatory cytokines and chemokines, as well as inducing macrophage migration and contributing to the pathogenesis of sepsis [11]. Injection of animals with high doses of LPS causes multiple organ failure, characterized by circulatory failure, systemic hypotension, hypo-reactivity to vasoconstrictors, subsequent problems with organ perfusion and the development of functional abnormalities [12], which reflect systemic inflammatory response syndrome and septic shock, rather than endotoxin-induced failure of lung, liver, and renal tissues [13].

Sepsis is the leading cause of death worldwide, with more than 750,000 cases of sepsis diagnosed annually and mortality rates ranging from 30 to 60%; this systemic inflammation accounts for approximately 200,000 deaths per year in the US alone [14]. Sepsis causes endothelial injury and neutrophil infiltration into tissues, leading to local injury, disturbed capillary blood flow and enhanced microvascular permeability, disseminated intravascular coagulation, circulatory collapse, hypoxia and, ultimately, multiple organ failure [15]. The aim of the current study was to investigate whether intravenous injection of honey can protect organs from lethal doses of LPS that induce sepsis in rabbits.

## 2. Results

### 2.1. Effect of Gelam Honey on Biochemical and Hematological Tests, Histopathology, and MPO Activity

**Figure 1:**
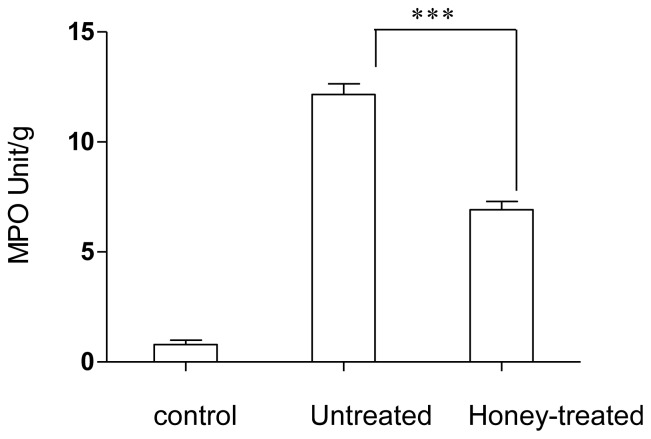
Effect of honey on neutrophil infiltration into lung tissues induced by a lethal dose of lipopolysaccharide (LPS). Myeloperoxidase (MPO) activity was measured in all groups (*n* = 6 per group) 8 h after LPS injection. MPO activity was significantly higher in the untreated (saline + LPS 0.5 mg/kg) group than in the treated (honey, 500 mg/kg + LPS 0.5 mg/kg) group. *** *P* < 0.002.

**Figure 2:**
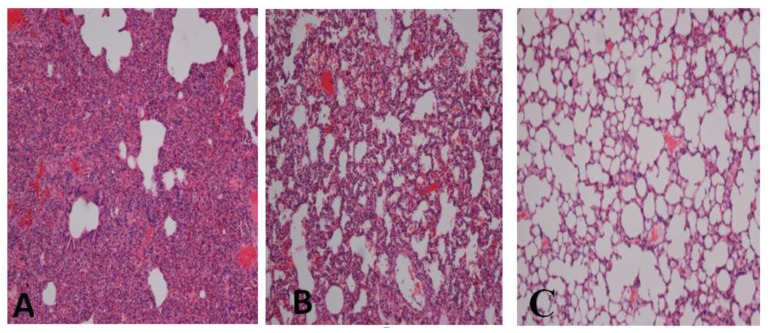
(**A**) Immune-cell infiltration and tissue damage in the lungs of rabbits from the untreated group 8 h after LPS injection; (**B**) Immune-cell infiltration and tissue damage in the lungs of rabbits from the honey-treated group 8 h after LPS injection; (**C**) Normal lung tissues in rabbits treated with saline. Hematoxylin and eosin staining; magnification 10×; scale bar, 30 μm.

**Figure 3:**
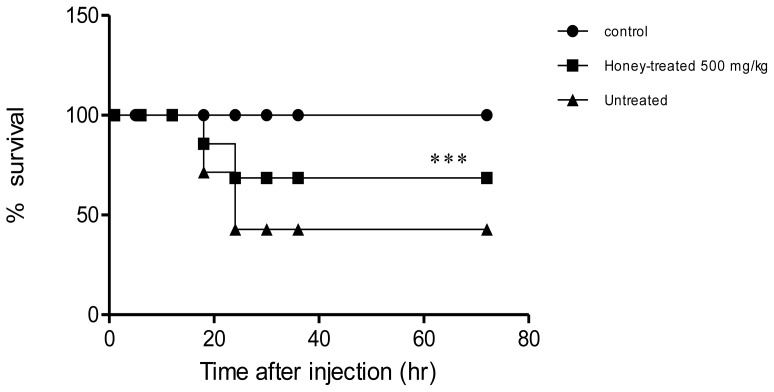
The effect of honey on the survival of rabbits injected with LPS (0.5 mg/kg). Rabbits in all three groups (*n* = 6 per group) received 1 mL injections of LPS into the ear vein. The survival rates in the untreated and honey-treated (60, 300, and 600 mg/kg) groups injected with 0.5 mg/kg LPS are shown as black triangles and black squares, respectively. Control rabbits received saline only (black circles). Honey was administered daily for 3 days after LPS treatment. Kaplan–Meier analysis showed significantly better survival rates in the honey-treated group (500 mg/kg + LPS) than in the untreated LPS group (LPS). *** *P* < 0.005.

Intravenous injection of honey resulted in potent protection against a lethal dose of LPS as evidenced by improved liver, kidney, cardiac and lipid profiles. Compared to the untreated group, the honey-treated group showed significant reductions in the levels of alanine transaminase (ALT), aspartate aminotransferase (AST), γ-glutamyltransferase (γ-GT), alkaline phosphatase (ALP), cholesterol, triglycerides, creatine kinase, creatinine, urea and amylase. Moreover, the honey-treated group showed higher RBC, WBC and thrombocyte counts than the untreated group (Table 1). Arterial blood gases and pH values were determined for all groups (Table 1). The honey-treated group showed mild respiratory alkalosis, while in the untreated group, the arterial blood pH was closer to acidosis. Blood pCO_2_ was lowered by LPS injection, but to the same level in the honey-treated group and untreated group, indicating that honey injection did not prevent the reduction in pCO_2_. Blood HCO_3_ and PO_2_ were higher in the honey-treated group than in the untreated group. There was clear evidence of hypoxia in the untreated group, as shown by the reduction in the pO_2_ value (Table 1). Neutrophil infiltration was reduced in the treated group; however, MPO activity in the honey-treated group was significantly lower than that in the untreated group (Figure 1). In addition, more histopathological changes were observed in the untreated group, as evidenced by cellular infiltration of the lungs (Figure 2). Finally, 66.7% of rabbits in the untreated group died compared with 33.3% in the treated group (Figure 3). Survival rates were monitored over a 2-week period.

## 3. Discussion

Previous studies have shown that honey has antioxidant, antimicrobial, and anti-inflammatory properties [16]. This study identified a protective role for honey against systemic damage induced by lethal doses of LPS in a rabbit model. These effects were evidenced by decreased blood chemistry parameters of organ dysfunction, decreased cellular infiltration into the tissues, and decreased mortality. To the best of our knowledge, this is the first study showing that honey can protect organs from lethal doses of LPS. The results indicate that honey can counteract the effects of LPS, which is a compound that can lead to organ and multi-organ failure.

When immune responses are insufficient, infections can lead to sepsis [17]. Many studies report that sepsis is a complicated pathophysiological and immunological process that causes alterations in the structure and characteristics of blood cells and tissues, leading to multi-organ failure. Lethal doses of LPS in animals induce a variety of organ and systemic changes that lead to organ failure and, ultimately, to death [18,19]. Previous studies have shown that the acute exposure of rabbits to LPS is associated with necrosis in organs such as the lungs and liver. The presence of polymorphonuclear leukocytes (PMNLs) was noted in association with necrosis in the lung and liver as well as an apoptotic cellular appearance in the LPS group. In addition, LPS stimulates the production of many cellular substances, such as cytokines, NO, vasoactive peptides, pro-coagulant factors, and prostaglandins, both *in vitro* and *in vivo* [15]. Earlier reports indicate that LPS and cytokines, such as TNF-α and IL-1β, induce apoptotic necrosis in cells and tissues [20,21]. Furthermore, LPS activates NF-κB, which activates many mediators including pro- and anti-inflammatory cytokines such as TNF-α, IL-1β, IL6 and IL-10 [22]. These cytokines enhance vascular permeability, stimulate the expression of adhesion molecules on endothelial cells, and induce infiltration of cells from the blood to tissues [23]. Sepsis-induced acute lung injury is a major clinical problem with significant morbidity and mortality [24–26]. PMNLs are thought to contribute significantly to the pathophysiologic features of acute lung injury [27–31]. A pathological hallmark of acute lung injury is subsequent tissue infiltration of neutrophils and pulmonary microvascular sequestration [32,33]. Enhanced pulmonary neutrophil sequestration and infiltration during sepsis changes the neutrophil profile by increasing neutrophil surface expression and activating cell-cell adhesion molecules, and enhancing the release of soluble mediators, production of cytokines, and generation of reactive oxygen species, NO, and ONOO^−^ [34–38]. Acute lung injury is characterized by increased MPO activity, a marker of neutrophil infiltration, increased expression and activity of cytokines and iNOS, high-protein pulmonary edema, and oxidant stress [31,39]. Pulmonary microvascular neutrophil sequestration and tissue infiltration are hallmarks of the pathogenesis of acute lung injury [33,40,41]. The present study is in agreement with previous studies showing that sepsis induces changes in pulmonary microvascular neutrophil sequestration and alveolar neutrophil infiltration, [34–36,42] as clearly shown in the untreated group but not in the honey-treated group (Figures 1 and 2). In addition, honey treatment decreased lung injury by inhibiting MPO activity. Therefore, as reported in our previous studies, honey may decrease lung injury through systemic inhibition of cytokines such as PGE_2_ and NO [9,10].

In this study, the reductions in RBC, WBC, and platelet counts observed in the untreated group confirm those seen in earlier reports [43,44]. Treatment with honey significantly attenuated the severe reductions in blood counts (WBC and RBC) and thrombocytopenia, suggesting that honey has a protective role against sepsis-induced disseminated intravascular coagulation. LPS causes disseminated intravascular coagulation, which is associated with coagulation disorders and loss of platelets. In the liver, LPS causes increases in AST, ALT, γ-GT, and lipid profiles [43,45–49], which are all markers of hepatic damage [44,49,50]. Our results confirm that sepsis caused liver failure, as shown by significantly elevated serum levels of AST, ALP, and γ-GT in the untreated group; honey inhibited these increases. Improved liver function tests after honey treatment indicate that honey may potentially protect liver cells from sepsis. Lipid profiles showed that cholesterol, triglycerides, and LDL levels were significantly increased in the LPS-induced untreated sepsis group but not in the honey-treated group. However, the HDL levels were significantly lower in the untreated group. Injection of LPS into animals induces renal dysfunction characterized by increased blood urea nitrogen and plasma creatinine levels [45,51]. Urea nitrogen and plasma creatinine levels were increased by LPS injection, but were lower in the honey-treated group than in the untreated group. Both our previous studies and the above results show that LPS increased the levels of hepatic damage markers, modified lipid metabolism, and increased lipid profiles, hematological values, and renal dysfunction [52]. Our results also show that Gelam honey protects organs from immune responses induced by lethal doses of LPS. Our previous study showed that Gelam honey contains many phenolic compounds with antioxidant and anti-inflammatory activity. In addition, its inhibitory effect on cytokines (TNF-α, IL-1β, and IL-10), high-mobility group protein 1 (HMG-1), and NO both *in vitro* and *in vivo* were studied [9,10,53]. Gelam honey also showed potent induction of HO-1, a molecule related to oxidative stress [53]. These activities, including the inhibition of cytokines and NO during severe sepsis, suggest that honey may be useful for the treatment of sepsis. The phenolic compounds in Gelam honey play a role in protecting tissues from LPS and free radicals due to their antioxidant activity, such as scavenging oxygen radicals, NO, and lipid radicals [54], and preventing cancer and various inflammatory disorders, such as arthritis and septic shock induced by endotoxemia [55–58]. The beneficial effects of honey, which include preventing histological changes and hypoxia in the organs of rabbits treated with LPS, may be directly related to its antioxidant activity, or indirectly related to the inhibition of PMNL chemotaxis, thereby preventing the production of the chemotactic agents implicated in tissue damage. We showed previously that Gelam honey has potent antioxidant activity and inhibits mediators of inflammation, such as cytokines, NO and PGE2 [9,10,53]. Allergic reactions constitute a potentially serious contraindication for injecting people with honey because honey contains bee-secreted and plant pollen-derived proteins that are known to induce allergic reactions [59].

## 4. Experimental Section

### 4.1. Preparation of Honey

Malaysian Gelam honey (*Melaleuca* spp.) was purchased from the department of Agriculture, Batu Pahat, Johor, Malaysia, and sent to Malaysian Nuclear Agency for sterilization using a Cobalt-60 source (Model JS10000). Honey was mixed with saline and filtered through a 0.20 μm syringe filter before injection.

### 4.2. Animals

Mice Balb/c mice (6–7 weeks of age) and New Zealand white male rabbits weighing 25 g and 2 kg, respectively, were kept in individual cages under standard conditions (12 h light and 12 h dark conditions); water and chow diet were available *ad libitum*. The study was carried out in accordance with the University of Malaya Animal Ethics Committee guidelines for animal experimentation and followed the approved protocols outlined in the project license (ANES/14/07/2010/MKAK (R)).

### 4.3. Toxicity Tests

The toxicity of Gelam honey was evaluated in mice (*n* = 8) for 1 month prior to the study. Four different doses of honey (10, 60, 300, and 600 mg/kg diluted in saline) were administered daily by injection into the tail vein (final volume, 100 μL). The control group received a similar volume of saline. Mice were observed for 3 h after injection. Symptoms and mortality were recorded for all groups. At the end of the study, all mice were sacrificed, and blood and organs were collected. Compared with the control group, the treated groups showed no abnormalities on biochemical and histopathological analysis of the liver, lungs, and kidneys (data not shown).

### 4.4. Induction of an Immune Response in Rabbits by LPS Stimulation and Treatment with Honey

New Zealand white male rabbits were divided into six groups (*N* = 6) of six animals (*n* = 6) and each group was treated as described below. An immune response was induced in four groups by intravenous injection of 0.5 mg/kg LPS (B: 0111; Sigma, St. Louis, MO, USA) diluted in saline. One hour before LPS injection, honey (500 mg/kg diluted in saline) was injected into the rabbits from two groups (treated group), while saline was injected into the rabbits from another two groups (untreated groups). The two remaining groups acted as negative controls and were given saline only and no LPS. All doses were administered in a final volume of 1 mL and were mixed immediately prior to injection. Three groups, one from each treatment stream, were used for biochemical and histopathological studies and assessment of myeloperoxidase (MPO) activity as described below, while the remaining three groups were used to assess survival rates. Survival was monitored every 12 h for 15 days.

### 4.5. Biochemical Analysis

Blood samples were collected from the ears of rabbits after 8 h of LPS injection. Serum was separated by centrifugation at 3000 × g at 23 °C, and hematological and biochemistry analysis were performed using an automated hematology cell counter analyzer (Sysmex XE-2100, Sysmex America, Inc.) and Advia 2400 Chemistry System (Siemen, Eschborn, Germany), respectively, in the clinical diagnostic laboratory at University of Malaya Medical Center. Biochemical analyses included measurement of glucose, liver, and kidney functions. The parameters used for hematological analysis were red blood cell count (RBC), white blood cell count (WBC), and platelet counts. Arterial blood samples were collected to measure pH, pO_2_, pCO_2_, and HCO_3_ using a blood gas analyser at the same time as the other biomedical tests were performed.

### 4.6. Myeloperoxidase Assay

Neutrophil infiltration into the lungs was monitored by measuring MPO activity as previously reported [60]. Briefly, tissue specimens were homogenized at 50 mg/mL in PBS (50 mM, pH 6.0) containing 0.5% exadecyltrimethylammonium bromide (Sigma-Aldrich). Samples were freeze-thawed three times and centrifuged at 13,000 rpm for 20 min. The supernatants were diluted 1:30 in assay buffer (50 mM PBS pH 6.0 containing 0.167 mg/mL *o*-dianisidine; (Sigma-Aldrich) and 0.0005% H_2_O_2_), and the colorimetric reaction was measured at 450 nm for between 1 and 3 min in a spectrophotometer (Microplate reader, Model 680, Life Science Research, Bio-Rad). MPO activity/g of wet tissue was calculated as follows: MPO activity (U/g wet tissue) = (A450) (13.5)/tissue weight (g), where A450 is the change in the absorbance of 450 nm light between 1 and 3 min after the initiation of the reaction. The coefficient 13.5 was empirically determined such that 1 U MPO activity corresponded to the amount of enzyme that reduced 1 μmol peroxide/min.

### 4.7. Histopathology

Liver, lung, heart, and kidney tissues were fixed in 10% formalin after the organs were dehydrated using graded ethanol solutions, cleared with xylene, paraffin embedded, sectioned, and stained with hematoxylin and eosin. Pathological changes were evaluated under a light microscope by a pathologist.

### 4.8. Statistical Analysis

All data are expressed as the mean ± confidence interval. Data were analysed using GraphPad prism statistical software (San Diego, CA, USA) for non-parametric analysis of variance. Kaplan–Meier analysis was used to compare survival rates. Differences were considered statistically significant at *P* < 0.05.

## 5. Conclusions

In summary, Gelam honey protects organs from lethal doses of LPS by improving organ functions, reducing infiltration by PMNs that cause tissue damage, reducing MPO activity and increasing the survival rate.

## Figures and Tables

**Table 1:** Assessment of organ damage in the control group and in honey-treated and untreated groups given a single intravenous injection of lipopolysaccharide.

Parameter	Normal Rabbits (*N* = 6)	Untreated (*N* = 6)	Honey-treated (*N* = 6)
Urea (mmol/L)	5.85 ± 0.20	55.85 ± 2.5	10.5 ± 0.23 a
Creatinine (mmol/L)	83.71 ± 2.5	154 ± 6.16	72 ± 2.87 a
ALT (IU/L)	54.125 ± 1.8	108.75 ± 3.6	78.75 ± 1.98 a
AST (IU/L)	27.75 ± 0.9	577.33 ± 19.2	231.5 ± 7.6 a
ALP (IU/L)	131.5 ± 4.1	542.75 ± 15.5	308.75 ± 11.4 a
GGT (IU/L)	10 ± 0.32	38.4 ± 0.6	25 ± 1.7 b
Triglyceride (mmol/L)	0.885 ± 0.03	10.434 ± 0.4	2.47 ± 0.13 a
Total cholesterol (mmol/L)	1.1125 ± 0.04	3.16 ± 0.1	1.8 ± 0.08 c
HDL (mmol/L)	0.65 ± 0.03	0.25 ± 0.006	0.545 ± 0.02 b
LDL (mmol/L)	0.202 ± 0.005	0.45 ± 0.01	0.32 ± 0.012
Creatine kinase (IU/L)	1327.4 ± 5.3	2168.3 ± 34	998.6 ± 26.8 a
pH (KPa)	7.38 ± 0.3	7.36 ± 0.3	7.5 ± 0.27 b
pCO_2_ (KPa)	4.3 ± 0.17	3 ± 0.12	3 ± 0.12
pO_2_ (KPa)	16.21 ± 0.53	7.65 ± 0.23	19.3 ± 0 .77 b
HCO_3_ (mmol/L)	19 ± 0.71	15 ± 0.51	19 ± 0.54 a
Platelet (10e9/L)	194.3 ± 6.4	144.5 ± 4.3	183.4 ± 7.6 b
Amylase (IU/L)	181.3 ± 7.2	215.5 ± 6.2	180 ± 5.2 b
RBC (10e12/L	9.27 ± 0.31	4.83 ± 0.15	8.3475 ± 0.32 a
WBC (10e9/L)	15 ± 0.6	6.05 ± 0.25	11.65 ± 0.36 a

a *P* < 0.001; significant effect of untreated group *vs.* honey-treated group;

b *P* < 0.003; significant effect of untreated group *vs.* honey-treated group;

c *P* < 0.005; significant effect of untreated group *vs.* honey-treated group.
